# An adjusted droplet digital PCR assay for quantification of vector copy number in CAR-T cell and TCR-T cell products

**DOI:** 10.1016/j.iotech.2024.101031

**Published:** 2024-12-04

**Authors:** J. Ma, S. Meyer, J. Olweus, C. Jin, D. Yu

**Affiliations:** 1 Department of Immunology, Genetics and Pathology, Uppsala University, Uppsala, Sweden; 2 Elicera Therapeutics AB, Gothenburg, Sweden; 3 Department of Cancer Immunology, Institute for Cancer Research, Oslo University Hospital Radiumhospitalet, Oslo, Norway; 4 Precision Immunotherapy Alliance, University of Oslo, Oslo, Norway

**Keywords:** droplet digital PCR (ddPCR), vector copy number (VCN), genetically engineered T cells

## Abstract

**Background:**

Genetically engineered T-cell therapy holds immense promise in cancer immunotherapy. These T-cell products are typically engineered by vectors that permanently integrate into the T-cell genome, thus raising concerns about potential risks of insertional mutagenesis. Therefore, it becomes imperative to assess the integrated vector copy number (VCN) as a critical safety parameter for gene-engineered cell products.

**Materials and methods:**

In this study, we developed a robust assay for assessing the VCN of chimeric antigen receptor-T cell and T-cell receptor T-cell products, based on the droplet digital polymerase chain reaction (ddPCR) method. To provide accurate representation of the VCN in gene-engineered cells, we implemented a calculation that factors in the putative transduction efficiency based on Poisson distribution statistics. The adjusted VCN value (VCN_adj_) was also compared with VCN value from sorted transgene-positive cell populations, to validate its accuracy.

**Results:**

This assay consistently and accurately determines the average VCN for cell products. By comparing the VCN in sorted transgene-positive cell populations, we validated the refinement calculation provides a closer approximation to the actual VCN within transduced cells, offering a more realistic representation of the VCN for engineered cell products.

**Conclusion:**

In summary, we present a reliable and robust ddPCR-based assay for quantification of VCN in gene-engineered cell products.

## Highlights

• Vector copy number (VCN) is a critical parameter for genetically engineered cell products.
• The study developed a ddPCR assay for quantifying VCN in CAR-T and TCR-T cell products.
• This assay consistently and accurately determines the average VCN in engineered cell products (VCN_bulk_).
• The refinement VCN_adj_ offers a more accurate representation of the VCN in engineered T-cell products.
• Validation against sorted transgene-positive cell populations confirms the accuracy of the adjustment calculation.

## Introduction

Genetically engineered T-cell therapy is promising due to its remarkable effectiveness and durable clinical responses.^1^^,^^2^ Two general approaches of engineered T-cell therapy have been developed, consisting of chimeric antigen receptor (CAR) T technology and T-cell receptor (TCR) T technology. CAR-T cells are engineered to express the synthetic CAR receptor on the cell surface, which enables the T cells to recognize and eliminate cancer cells expressing the targeted surface antigen. In contrast, TCR engineered T cells use a natural/slightly modified TCR to recognize the tumor-specific epitopes presented on the major histocompatibility complex molecules on the surface of the tumor cells.

Viral vectors, often replication-incompetent γ-retroviral or lentiviral vectors, serve as carriers for introducing transgenes encoding CAR molecules. The transduction process leads to stable integration of transgenes into the cellular genome. The quantity of integrated transgenes within the cellular genome is positively correlated to the efficacy of CAR-T cells in clinical applications.^3^ An increase in the copy number of integrated transgene [vector copy number (VCN)], however, is also paralleled by a higher risk of uncontrolled integration, potentially causing insertional mutagenesis by disrupting nearby host gene transcription.^4^ Even though secondary malignancies were reported for patients who received gene-engineered cell therapies,^5^ the risk of secondary malignancy is regarded low and not significantly different from patients who received standard-of-care.^6^^,^^7^ A product with higher VCN might potentially increase the genotoxicity and therefore, however, careful monitoring of integrating VCN is crucial to determine the VCN levels that confer desirable clinical efficacy while minimizing the genotoxic and oncogenic potential of viral vectors, as required by regulatory authorities before the release of a drug product.^8^^,^^9^

Traditionally, quantitative real-time polymerase chain reaction (qPCR) was used for VCN measurement. This method relies on a standard curve for quantification and offers limited precision.^10^ More recently, methods based on droplet digital PCR (ddPCR) that directly quantify the absolute counts of target have been developed for VCN measurement and CAR-T cell monitoring after clinical treatment.^3^^,^^11^, ^12^, ^13^, ^14^, ^15^ The ddPCR-based method provides improved resolution, precision, and reproducibility. Most of these established assays, however, examine and report VCN of the bulk CAR-T cell product, even though only a fraction (20%-70%) of the cells are transduced cells. This results in an underestimation of the VCN for the actual transduced cells.

In the present study, we report the development of a ddPCR assay for precise VCN quantification in CAR-T cell and TCR-T cell products. Moreover, we implement a simple refinement step to reflect VCN more accurately for the transduced cells. Our results demonstrate that this assay maintains high levels of specificity, precision, and reproducibility, making it a valuable tool for advancing engineered T-cell therapy research and development.

## Materials and methods

### Human peripheral blood mononuclear cells

Human peripheral blood mononuclear cells (PBMCs) were isolated by Ficoll-Paque (GE Healthcare Life Science, Uppsala, SE) gradient separation from buffy coats of healthy anonymized donors, obtained from the blood center at Uppsala University Hospital. The isolated PBMCs were cryopreserved in solution containing 90% fetal bovine serum (Gibco, Bleiswijk, NL) and 10% dimethyl sulfoxide (Sigma, Solna, SE) at a density of 50 ×  10^6^ cells per milliliter and stored at −150°C until further use.

### Genetically engineered CAR-T and TCR-T cell production

CAR-T cells were produced on the CliniMACS Prodigy in a 13-day manufacturing protocol. The frozen PBMCs were thawed and washed following activation with TransACT (Miltenyi Biotec, Teterow, DE) for 48 h. Cells were then transduced with γ-retroviral vectors (BioNTech IMFS, Mainz, DE) encoding for either CAR20 (targeting CD20) or CARIL13R (targeting IL13Ra2)^16^ at multiplicity of infection (MOI) of 5. Both CARs contain the 4-1BB (CD137) and CD3zeta signaling domains.^17^

TCR-engineered CD8 T cells were produced on the CliniMACS Prodigy in a 10-day manufacturing protocol. T cells from a cryopreserved apheresis product were enriched through positive selection with CD4 and CD8 beads (1 : 20 CD4/CD8 bead ratio), activated for 48 h with TransACT (Miltenyi Biotec) and transduced with a γ-retroviral vector (BioNTech IMFS) encoding for the chimeric TdT TCR^18^ at MOI 4.

Both CAR-T and TCR-T cells were cryopreserved after harvest. The final cell products were analyzed for VCN.

### Genomic DNA extraction and quantification

Genomic DNA (gDNA) was isolated from cells using the QIAamp DNA Mini Blood Kit (Qiagen, Kista, SE), following the manufacturer’s instruction. The quantity of extracted gDNA was determined on the spectrophotometer (NanoDrop 1000, Thermo Fisher Scientific, Bleiswijk, NL). Isolated DNA was stored at −20°C until use.

### Design of PCR primers and probes

The ddPCR reaction was settled as a duplex assay to simultaneously quantify the copy number of inserted retroviral vector and human reference gene *telomerase reverse transcriptase* (*TERT*).

**Figure 1:**
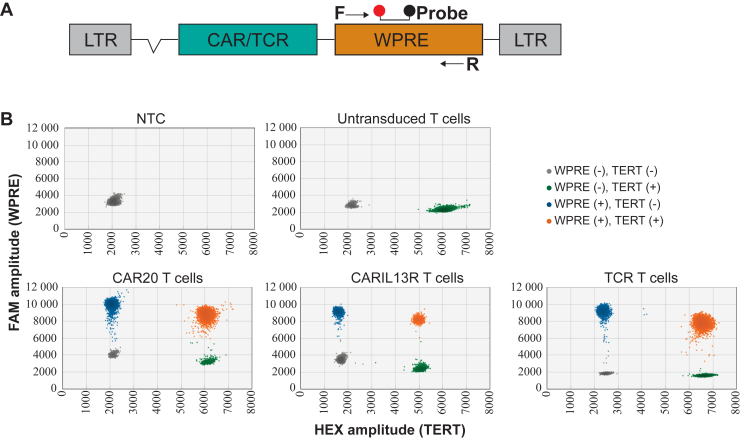
**Assay specificity.** (A) CAR/TCR retroviral vector map with highlighted location of target sequence (WPRE). Lines with arrows are forward (F) and reverse (R) primers used to span the target sequence. Amplicons are detected by a FAM (with quencher)-labeled probe. (B) Specificity of the established ddPCR assay for unique detection of retroviral vector engineered CAR-T and TCR-T cells. A representative example of a two-dimensional (2D) plot is shown. X-axis, fluorescence intensity detected in the HEX channel (reference gene *TERT*); Y-axis, fluorescence intensity detected in the FAM channel (WPRE element). The grey cluster represents the FAM^−^HEX^−^ droplets, containing no WPRE and no reference copies. The blue cluster represents FAM^+^HEX^−^ droplets, containing only WPRE copies. The green cluster represents FAM^−^HEX^+^ droplets, containing only reference copies. The orange cluster represents FAM^+^HEX^+^ droplets, containing both WPRE and reference copies. The no-template control (NTC) only appeared as a grey cluster and the non-transduced sample showed the grey and green cluster. The engineered CAR-T and TCR-T cells showed all four clusters. CAR, chimeric antigen receptor; ddPCR, droplet digital polymerase chain reaction; FAM, fluorescein amidite; HEX, hexachlorofluorescein; LTR, long terminal repeat; TCR, T-cell receptor; TERT, telomerase reverse transcriptase; WPRE, woodchuck posttranscriptional regulatory element.

To quantify retroviral vector insertion, the primer pair and probe were designed to specifically target the woodchuck posttranscriptional regulatory element (WPRE),^19^, ^20^, ^21^, ^22^ which is commonly present in a γ-retroviral or lentiviral vector at the 3′-untranslated region of the transgene (Figure 1). Therefore, WPRE can serve as a surrogate marker for detection of the inserted vector, and it can be applied to different products. The amplicon is confirmed to lack the EcoRI restriction enzyme recognition site. This WPRE primer pair and probe were ordered from Bio-Rad Laboratories (Solna, SE) as custom assay, wherein the probe is labelled by the FAM fluorescent dye. The primer pair and probe targeting the reference gene *TERT* was predesigned by Bio-Rad (Assay dHsaCP2500351, Bio-Rad Laboratories), wherein the probe for *TERT* is labelled by the HEX fluorescent dye.

The annealing temperature was optimized to ensure the optimal assay performance. CAR-T cell products were used as positive control, while non-transduced cells served as negative control. Droplet digital PCR (ddPCR) reactions were carried out across an annealing temperature gradient with eight intervals from 50°C to 65°C. Based on separation between positive and negative droplets in the FAM and HEX channel, the optimal annealing temperature was fixed to 58°C and subsequently used for ddPCR runs.

### Droplet digital PCR

The general Bio-Rad guideline for ddPCR was followed. For each 20 μl ddPCR reaction, ddPCR Supermix for Probes (No dUTP) (1×) was mixed with 50 ng gDNA or 5 μl plasmid DNA, 900 nM of each primer, 250 nM probe, and 5 U EcoRI restriction enzyme (Thermo Fisher). The reaction mix was incubated at room temperature (20-25°C) for 5 min, and the droplets were generated using a QX200™ Manual Droplet Generator (Bio-Rad Laboratories) according to the manufacturer’s instruction. The emulsion droplets were transferred to a 96-well ddPCR plate. Standard PCR amplification was carried out in a C1000 Thermal Cycler (Bio-Rad) as: pre-denaturation at 95°C for 10 min, 40 amplification cycles with 94°C for 30 s and 58°C for 1 min, and a final enzyme deactivation step at 98°C for 10 min. Temperature ramp rate was set as 2°C per second. After PCR amplification, droplets were analyzed in a QX200 Droplet Reader with QX Manager 2.0 software (Bio-Rad Laboratories).

The copy numbers for WPRE element and reference gene *TERT* were determined using QX Manager software (Bio-Rad Laboratories). Assuming that each diploid cell contains two copies of the *TERT* gene, the average VCN (VCN per cell, termed as VCN_bulk_) for the bulk sample was calculated as the ratio between the copy number of the targeted *WPRE* element and the reference *TERT* gene, according to the following formula:

$${VCN}_{bulk}=\frac{\text{WPRE}\;\text{copy}\;\text{number}}{\text{TRET}\;\text{copy}\;\text{number}}\times 2$$

### The lower limit for detection and quantification of WPRE

The retroviral vector plasmid encoding the CAR and WPRE sequence was used to determine the detection and quantification limit for WPRE. A serial dilution (1 : 2) of this plasmid was freshly prepared before each ddPCR assay. The DNA concentration of the undiluted sample was quantified with a Qubit Fluorometer using a double-stranded DNA (dsDNA) broad-range (BR) kit (Invitrogen, Uppsala, SE). The copy number was calculated based on DNA molecular weight and further calculated for the serially diluted samples. The diluted samples ranging from 2500 copies to 1 copy/μl, subject to ddPCR reaction mix, were further analysis by ddPCR. No-template controls (NTCs) were used to determine the limit of blank (LoB). The LoB, the lower limit of detection (LLoD), and the lower limit of quantification (LLoQ) were calculated using the following equations^23^:

$$LoB={Mean}_{blank}+1.645\left({SD}_{blank}\right)$$

$$LLoD=\mathrm{MAX}\left[LoB+3.3\left({SD}_{blank}\right),lowest\;dilution\;tested\right]$$

$$LLoQ=\mathrm{MAX}\;\left[LoB+10\left({SD}_{blank}\right),lowest\;dilution\;tested\right]$$

### The precision and linearity of ddPCR-based VCN assay

**Figure 2:**
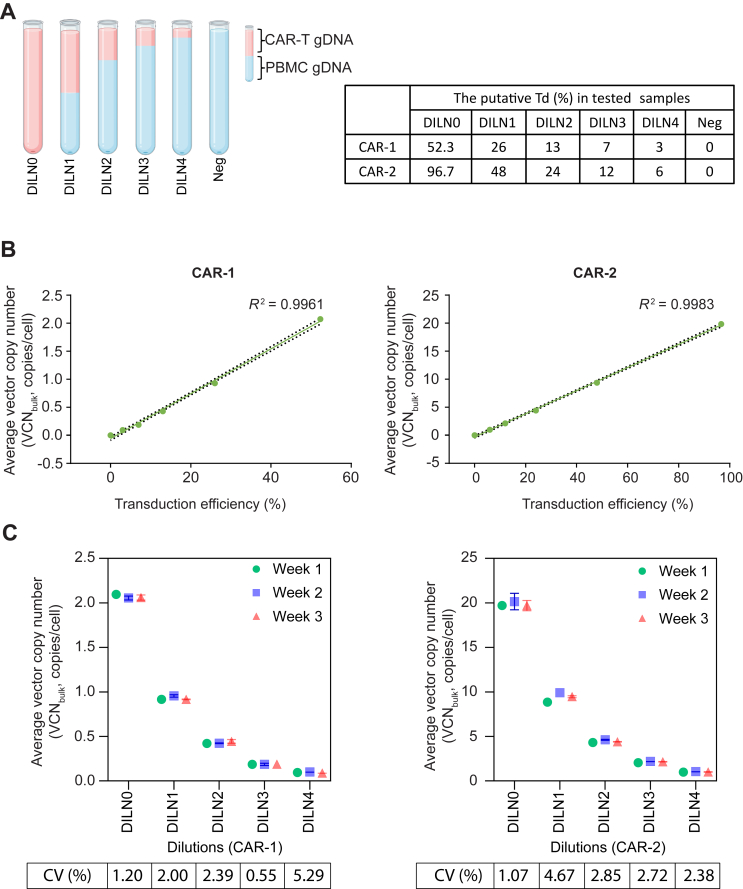
**Determination of VCN in CAR-T cell products and the inter-assay repeatability.** (A) Schematic illustration of spike sample setup. Genomic DNA (gDNA) from CAR-T cell products was twofold serially diluted with gDNA isolated from PBMC (same donor). Genomic DNA (gDNA) was isolated from CAR-T cell products (CAR-1 and CAR-2) and non-engineered PBMCs from the same two healthy donors. DILN0 represents undiluted samples, and DILN1-DILN4 corresponds to samples with 1 : 2, 1 : 4, 1 : 8, and 1 : 16 dilutions, respectively. The negative control (Neg) consisted of gDNA isolated from PBMCs. The putative transduction efficiency is shown in the table (right), as calculated based on the measurement of the undiluted sample using flow cytometry. (B) The diluted samples were analyzed by ddPCR to determine the average VCN (VCN_bulk_). The VCN_bulk_ of diluted samples from CAR-1 (left) and CAR-2 (right) was plotted against putative transduction efficiency and fitted with a linear regression curve. The data represent the mean values of three independent experiments. (C) Assessment of repeatability. The diluted samples from CAR-1 (left) and CAR-2 (right) were tested at three different time points with ∼1-week interval. The experimental setup was carried out by the same person using the same instrument. Coefficients of variation (CV%) between each repeats are presented below each dilution conditions. The *P* value of two-way analysis of variance comparing average VCN per sample was >0.05. CAR, chimeric antigen receptor; ddPCR, droplet digital polymerase chain reaction; PBMCs, peripheral blood mononuclear cells; VCN, vector copy number.

To validate the ability of the established assay to distinguish detected signal from noise background, the gDNA from two CAR-T cell products (CAR-1 and CAR-2) were serial diluted and spiked into the same amount of human gDNA isolated from PBMCs derived from the same donor as the CAR-T cell product (Figure 2). A sample containing gDNA isolated from non-transduced PBMCs (without WPRE element) was used as negative control.

### Cell sorting

The cells were labeled with LIVE/DEAD^TM^ fixable Aqua dye (Thermo Fisher), followed by surface marker antibodies staining (CD3 and CAR) before fluorescence-activated cell sorting (FACS). The CD3^+^CAR^+^ T cells (transduced cells) and CD3^+^CAR^−^ T cells (non-transduced cells) were sorted on a BD FACS Melody (BD Biosciences, Stockholm, SE). Debris was excluded by adjusting the threshold setting and applying a scatter gate during sorting.

### Refinement calculations

To normalize the VCN according to the percentage of gene-modified cells, the adjusted VCN (VCN_adj_) was calculated with the formula:

$${VCN}_{adj}={VCN}_{bulk}/\left(1-e^{-{VCN}_{bulk}}\right)$$

where VCN_bulk_ is the average VCN in bulk CAR-T cell product as determined by ddPCR, and 1-e^−VCNbulk^ is the putative transduction efficiency calculated based on Poisson distribution.

## Results

### Assay specificity

Primers and the probe were designed to specifically target the WPRE region, which is commonly used to increase the transgene expression in viral vectors^19^, ^20^, ^21^, ^22^ but absent from the human genome (Figure 1A). Firstly, we confirmed the specificity of the primer-probe set for different WPRE-containing CAR-T and TCR-T cell products developed in our laboratories (Figure 1B). WPRE-positive (FAM) droplets were only detected in the transduced cell products (blue/orange dots, Figure 1B, lower panel), while no FAM-positive droplets were detected in non-transduced control T cells (Figure 1B, upper panel). The *TERT* reference gene (HEX), however, was detected in all samples except the NTC. Further, the clear separation between positive and negative droplets (Figure 1B) demonstrates the assay’s specificity.

### Assay accuracy and sensitivity for WPRE quantification

**Figure 3:**
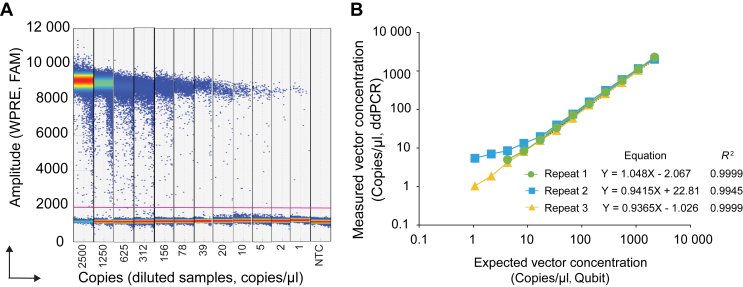
**Sensitivity and accuracy assessment.** A plasmid encoding the WPRE sequence was serially diluted and assessed in the ddPCR assay. (A) The 1D plot (FAM, WPRE) shows the amplitude of each diluted sample (range: 2500-1 copies/μl). No-template control (NTC) served as negative control. Positive and negative droplets were delimited by the threshold (pink line). (B) The vector concentration measured by ddPCR was plotted against the putative vector concentration measured by Qubit quantification. The expected vector concentration is shown on the X-axis, while the measured vector concentration is indicated on the Y-axis. Linear regression curves with equation and the coefficient of determination (*R*^2^) were calculated for each of the three independent experiments. ddPCR, droplet digital polymerase chain reaction; FAM, fluorescein amidite; WPRE, woodchuck posttranscriptional regulatory element.

Due to lack of reference material with known copy number of WPRE, it becomes challenging to directly assess the accuracy of this assay. We therefore opted to use a plasmid containing the WPRE sequence to evaluate the accuracy. We assessed the linearity performance on twofold serially diluted samples, ranging from 2500 copies down to 1 copy/μl, by ddPCR in triplicate, at three different time points with 1-week intervals. As illustrated by the 1D amplitude plot, we achieved a good distinction between positive and negative droplets (Figure 3A). A log-log best fit line showed good linearity between the expected vector concentration (measured by Qubit) and the measured vector concentration (ddPCR) with an average *R*^2^ value of 0.9981 (0.9999, 0.9945, and 0.9999 for the three repeats, respectively) (Figure 3B). These results indicate that high specificity and accuracy can be achieved with the current design for quantifying the WPRE sequence.

In each ddPCR run, nuclease-free water was used as NTC. This led to the determination of the LoB for WPRE detection as 1.3 copies/μl of the reaction mix. Subsequently, we calculated the LLoD to be 2.3 copies/μl of the reaction mix, while the LLoQ was established at 6.4 copies/μl of the reaction mix.

### VCN quantification and the inter-assay repeatability

Assay repeatability and reproducibility were assessed on CAR20-T cell products generated from two different donors with a retroviral vector containing the WPRE sequence, designated as CAR-1 and CAR-2. The CAR transduction efficiency measured by flow cytometry differed between the two CAR-T cell products, with CAR-1 at 52.3% and CAR-2 at 96.7%. The gDNA of each sample was isolated, and twofold serially diluted with a fixed amount of non-template background DNA (human gDNA isolated from PBMCs derived from the same donor as used for the CAR-T cell product manufacturing) (Figure 2A). Addition of gDNA is to rule out the matrix interference and mimic situations of low copy number in test samples. Notably, the concentration values of the WPRE sequence in all diluted samples exceeded the LLoQ (6.4 copies/μl) (Supplementary Table S1, available at https://doi.org/10.1016/j.iotech.2024.101031), confirming that the values of all sample wells were reliable. Furthermore, a strong linearity was observed between the measured average VCN values (VCN_bulk_) and expected transduction efficiency in both CAR-T cell products (*R*^2^ = 0.9961 for CAR-1 and *R*^2^ = 0.9983 for CAR-2) (Figure 2B). These data demonstrate the fidelity of the established ddPCR assay, as a reduction in the amount of gDNA from CAR-T cells in the sample led to a proportional decrease of the average VCN.

To assess inter-assay repeatability, the diluted samples were analyzed by ddPCR at three different time points with 1-week intervals. The same operator carried out all tests using the same instruments. Two-way analysis of variance comparing the measured VCN across time revealed no statistically significant differences (*P* > 0.05) (Figure 2C). The inter-assay coefficients of variation (CV, %) were consistently <6% (Figure 2C), indicating a high level of repeatability of the established assay. The mean values of VCN_bulk_, standard deviation, and %CV values for diluted samples are presented in Supplementary Table S2, available at https://doi.org/10.1016/j.iotech.2024.101031. These results demonstrate the feasibility of using ddPCR for the determination of the average VCN for CAR-T cell products transduced with a vector simultaneously encoding the WPRE target sequence with high repeatability.

### Refinement of the VCN for transduced cells

One notable limitation of the assay is its reliance on the average VCN within a bulk cell population that includes both transduced and non-transduced cells. This is particularly problematic in CAR-T cell products with low transduction efficiency, where the VCN for the transduced cells can be significantly underestimated. To improve the reliability and representativeness of VCN as a safety metric for clinical cell products, it is crucial to specifically quantify the VCN within the transduced cells.

**Figure 4:**
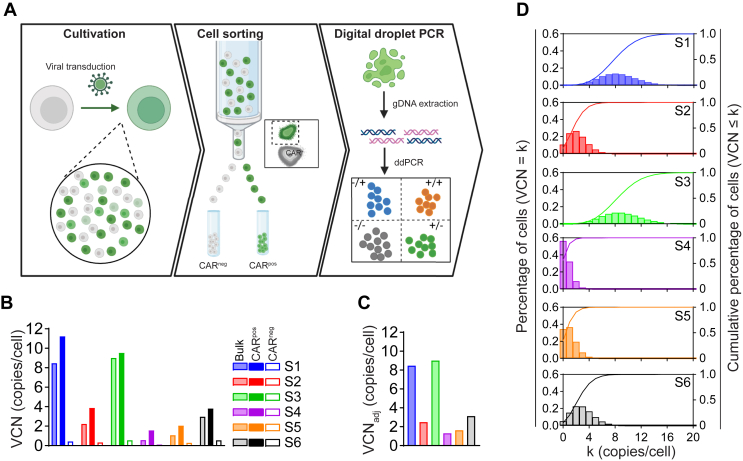
**The adjusted VCN in CAR-T cell products.** (A) Workflow for cell sorting and ddPCR. CAR-T cell products were analyzed by flow cytometry to determine the transduction efficiency and sorted into CAR-positive and CAR-negative populations. After isolation of genomic DNA (gDNA) from the sorted cells, samples were analyzed by ddPCR to determine the VCN. (B) The VCN value determined on the bulk cell populations, sorted CARneg, and CARpos cell populations. (C) The adjusted VCN_adj_ normalized against theoretical transduction efficiency based on Poisson statistics. (D) Probability and cumulative distribution for each sample. The percentage of cells at a given VCN is plotted as bar on the left Y-axis and the cumulative percentage of cells at a VCN smaller and equal to k is plotted as curve on the right Y-axis. The connective curve is for visualization; it is defined only when k is integers. Calculations were based on the probability mass function and cumulative distribution function. CAR, chimeric antigen receptor; ddPCR, droplet digital polymerase chain reaction; VCN, vector copy number.

We try to adjust the bulk VCN according to theoretical transduction efficiency based on Poisson distribution statistics, which presented the best estimate when the actual transduction rate is unknown.^24^^,^^25^ We set out to evaluate the feasibility and accuracy of this approach by sorting CAR-positive (CARpos) and CAR-negative (CARneg) cells from six CAR20-T cell products (Figure 4A), each exhibiting different transduction efficiencies and average VCNs. In our analysis, we discovered that the CARneg populations consistently showed a VCN of less than one copy per cell (Figure 4B and Table 1). This finding aligned perfectly with our sorting process, confirming the low/no transduction in these fractions. In contrast, the sorted CARpos cells exhibited a notably higher VCN (VCN_CARpos_) compared with the average VCN (VCN_bulk_) of the same samples, especially in cases where VCN_bulk_ is low. For instance, the comparisons revealed VCN levels such as 0.57 versus 1.57 for sample S4, demonstrating the inadequacy of relying on average VCN alone to represent the transduced cell population accurately.

**Table 1:** VCN in bulk and sorted cells

Samples	S1	S2	S3	S4	S5	S6
VCN_CARneg_	0.43	0.32	0.55	0.11	0.30	0.54
VCN_CARpos_	11.22	3.87	9.53	1.57	2.04	3.81
VCN_bulk_	8.47	2.23	9.00	0.57	1.07	2.97
VCN_adj_	8.47	2.50	9.00	1.31	1.63	3.13
Transduction % (by flow)	78.6	52.3	91.5	27.2	37.5	66.2

CAR, chimeric antigen receptor; VCN, vector copy number.

The adjusted VCN value (designated as VCN_adj_), however, is more appropriate in representing the VCN of transduced cells (Figure 4C and Table 1) since the values are closer to VCN_CARpos,_ especially in cases with low VCN_bulk_. We also estimated the percentage of cells at each given VCN level and assessed the cumulative distribution of cells with a VCN less than or equal to a given value (Figure 4D). The results revealed that a substantial proportion of cells displayed higher VCN levels when the VCN_adj_ was elevated. For example, in samples S1 and S3, where VCN_adj_ was 8.47 and 9, approximately half of the cells had a VCN exceeding 9. Conversely, in samples with lower VCN_adj_ values (<5), >90% of cells had a VCN <5.

Notably, the calculated theoretical percentage of untransduced cells (VCN of zero) is consistently lower than the values obtained through flow cytometry analysis, suggesting that some cells may not express CAR at detectable levels but still contain viral vectors. This is also in line with our observation that we can still detect viral vectors in the sorted CARneg population despite their lack of detectable CAR expression.

In summary, our findings strongly support the feasibility of using VCN_adj_ as a reliable metric for cell products, alongside the percentage of cells at given VCN levels and their cumulative distribution. This comprehensive approach not only enhances the accuracy of VCN reporting, but also provides deeper insights into the characteristics of CAR-T cell products.

## Discussion

Stable engineering of T cells with a therapeutic CAR or TCR is achieved by viral vectors that have the capability to integrate into the host cell gDNA. Genomic integration also leads to the potential risk of insertional mutagenesis, and recent reports of rare cases of secondary malignancies warrant careful monitoring to identify risk factors, which might relate to high VCN. Therefore, controlling the insertional VCN in these products before their release is important, calling for rapid and accurate assays.

Quantitative real-time PCR (qPCR) is a widely used method for VCN determination.^26^ More recently, methods based on ddPCR have emerged, offering improved sensitivity and accuracy.^15^^,^^26^, ^27^, ^28^, ^29^, ^30^ In addition, ddPCR-based methods require less input DNA per reaction (only 20 ng DNA) compared with qPCR-based methods.^11^^,^^15^ In this study, we established and validated a ddPCR assay for determination of the VCN in CAR-T and TCR-T cell products. The assay was designed to detect the WPRE sequence, often used in viral vector designs^19^, ^20^, ^21^, ^22^ and is thus suitable for the VCN quantification, e.g. viral vector engineered CAR-T cell products. The assay demonstrated excellent accuracy and sensitivity, with a low CV value (<6%) across different time points, indicating high inter-assay precision. Therefore, the method proves to be suitable for VCN quantification for clinical T-cell products such as CAR-T.

During clinical trials, monitoring the kinetics of CAR-T cells is another important aspect, as CAR-T cell *in vivo* expansion and long-term functional persistence are correlated with the treatment efficacy and toxicity.^31^, ^32^, ^33^ We anticipate that this assay can also be used for CAR-T cell monitoring in patients after treatment to follow up the pharmacokinetics. Additional validation work, however, should be carried out before its application in this context.

In this study, we choose to use a pre-established assay to quantify reference gene (*TERT*) without validation. The copy number of *TERT* may vary in cancerous cells depending on the clinical context, with *TERT* amplification in ∼2% of cases across a pan-cancer analysis.^34^ We think the risk of amplified *TERT* in healthy T cells is low, however, since stringent criteria will be applied to the final CAR-/TCR-T cell therapy products to exclude cancerous cells. Our method specifically quantifies WPRE gene in gamma-retroviral vector engineered cell products. In theory, we believe that this method principle can be also applied for lentiviral vector transduced cell products, as well as for cell products lacking the WPRE gene inserts. In the latter case, screening and validation of a new set of primer-probes is needed.

VCN quantification of the average value for the entire population of tested cells can potentially lead to the underestimation of the actual VCN within transduced cells. One approach to determine the actual VCN is the analysis of sorted CAR-expressing cells. This, however, adds complexity and workload. More importantly, not all transduced (viral genome-containing) cells express the CAR molecules at a level that can be detected and sorted. To circumvent these challenges, we established a simple method for the approximation of the actual VCN in transduced cells by normalizing the average VCN (VCN_bulk_) against theoretical transduction efficiency calculated using Poisson statistics. Applying Poisson statistics for VCN calculation were also reported by Fehse et al.^24^^,^^25^ The calculation also had its own limitations as it is purely based on probability theory and all mathematical and biological assumptions which include (i) each transduction is random and independent; (ii) all cells are equally susceptible to transduction; (iii) all cells grow at a similar rate regardless of transduction and insertional copies. Importantly, we also experimentally verified and justified the mathematical refinement of VCN (VCN_adj_) as biologically appropriate since the VCN_adj_ value falls in the range between VCN_b__ulk_ and VCN_CARpos_, given the fact that there can be viral vector-containing cells present in the CARneg population as well. Non-transduced cells (as determined by flow cytometry analysis) had an average VCN <1, confirming low/no transgene integration into these cells.

To be noticed, our assay assesses the VCN at the population level and thus provides an average value, which implies that there will be a certain percentage of cells with a higher VCN than the average value. Santeramo et al.^35^ explored the VCN distribution at single-cell level, and demonstrated that the distribution of single-cell VCN follows the average of population VCNs. Single-cell VCN workflows require more resources and are more labor demanding, however, making them less suitable for standard measurements on drug products. In fact, we use Poisson statistics and present theoretical probability distribution of cells at each given VCN as well as their accumulative distributions. We think this information is as important as the average VCN value to understand the overall VCN distribution within the cell product.

In conclusion, we present here a method for rapid and precise VCN quantification. Analyzing the bulk cell population and refining the value through normalization (VCN_adj_) is both adequate and cost-effective for approximation of the VCN in transduced cells in, for example, the CAR-T cell drug products, finding a balance between accuracy and practicality.
