# Supplementary material for: An adjusted droplet digital PCR assay for quantification of vector copy number in CAR-T cell and TCR-T cell products
Source: Immunooncol Technol. 2024 Dec 4;25:101031. doi: 10.1016/j.iotech.2024.101031 (PMC11997555; doi:10.1016/j.iotech.2024.101031)
Supplement: Supplementary Material [file mmc1.docx]

Table S1. Absolute copy number of WPRE gene in diluted samples

| **Samples** | | **WPRE concentration (copies/μL)** | | |
| --- | --- | --- | --- | --- |
|  |  | **Repeat 1** | **Repeat 2** | **Repeat 3** |
| CAR-1 | DILN0 | 519 | 531 | 497 |
|  | DILN1 | 249 | 252 | 236 |
|  | DILN2 | 114 | 115 | 112 |
|  | DILN3 | 48 | 49.8 | 48 |
|  | DILN4 | 25 | 26.2 | 21.9 |
| CAR-2 | DILN0 | 5286 | 6011 | 4996 |
|  | DILN1 | 2398 | 2818 | 2322 |
|  | DILN2 | 1241 | 1320 | 1135 |
|  | DILN3 | 600 | 634 | 562 |
|  | DILN4 | 306 | 306 | 271 |

Table S2. Inter-assay analysis

| **Samples** | | **Mean** | **SD** | **CV%** |
| --- | --- | --- | --- | --- |
| CAR-1 | DILN0 | 2.075 | 0.0248 | 1.197 |
|  | DILN1 | 0.9295 | 0.0184 | 1.978 |
|  | DILN2 | 0.430 | 0.0103 | 2.389 |
|  | DILN3 | 0.187 | 0.0010 | 0.659 |
|  | DILN4 | 0.094 | 0.0050 | 5.293 |
| CAR-2 | DILN0 | 19.850 | 0.2121 | 1.069 |
|  | DILN1 | 9.405 | 0.4389 | 4.666 |
|  | DILN2 | 4.447 | 0.1268 | 2.853 |
|  | DILN3 | 2.130 | 0.0579 | 2.717 |
|  | DILN4 | 1.014 | 0.0241 | 2.380 |
